# Three-dimensional reconstructive kidney volume analyses according to the endophytic degree of tumors during open partial or radical nephrectomy

**DOI:** 10.1590/S1677-5538.IBJU.2014.0417

**Published:** 2016

**Authors:** Dong Soo Park, Young Kwon Hong, Seung Ryeol Lee, Jin Ho Hwang, Moon Hyung Kang, Jong Jin Oh

**Affiliations:** 1 Department of Urology, CHA Bundang Medical Center, CHA University, Seongnam, Korea;; 2 Department of Urology, Seoul National University Bundang Hospital, Seongnam, Korea

**Keywords:** Kidney, Nephrectomy, Surgical Procedures, Operative, Neoplasms

## Abstract

**Objectives:**

To investigate the renal function outcomes and contralateral kidney volume change measured by using a 3-dimensional reconstructive method after open partial nephrectomy (PN) or open radical nephrectomy (RN) according to the endophytic degree of tumors.

**Materials and Methods:**

We included 214 PN and 220 RN patients. According to the endophytic degree of the tumors, we divided patients into 3 groups. Patients were assessed for renal function and kidney volume change both preoperatively and postoperatively at 6 months. Kidney volume was calculated by using personal computer-based software. Subgroup analyses was performed for tumor >4cm.

**Results:**

Larger and complex tumors were more frequent in the RN group than PN group. Among patients with exophytic and mild endophytic tumors, the mean postoperative renal function was well preserved in PN group and the mean contralateral kidney volume significantly increased in the RN compared to the PN group (PN, 145.55 to 149.98mL; 3.0% versus RN, 143.93 to 169.64mL;17.9% p=0.006). However, in fully endophytic tumors, compensatory hypertrophy of the contralateral kidney was similar between PN and RN (PN, 138.16 to 159.64mL; 15.5 % versus RN, 138.65 to 168.04mL; 21.2% p=0.416) and renal functional outcomes were similar between both groups. These results were also confirmed in tumors >4cm in size.

**Conclusions:**

In fully endophytic tumors, especially large tumors, the postoperative renal function and contralateral kidney volume were similar; therefore, we should consider RN preferentially as surgical option for these tumors.

## INTRODUCTION

Partial nephrectomy (PN) is currently the standard procedure for surgical treatment of small renal cortical tumors, especially clinical T1a tumors (<4cm) (1, 2). For clinical T1b renal tumors, (≥4cm), elective PN is occasionally recommended to be performed in high volume centers, since present equivalent oncological results to radical nephrectomy (RN) and superior renal functional preservation (3, 4). Even in renal tumors ≥7cm, some reports showed remarkable results with acceptable complication rates and with oncologic outcomes comparable to RN. PN in these large sized tumors could preserve the renal function (5).

However, actual renal functional preservation after PN has not been defined in a standard manner in the literature. Currently, kidney volume is believed to a measurable parameter to predict renal function. One autopsy study showed kidney volume strongly correlated with the number of functional nephrons (6); kidney volume during donor nephrectomy was correlated with renal function in living donor (7, 8), and Jeon et al. (9) showed that preoperative kidney volume is an independent predictor of renal function in renal cell carcinoma (RCC) patients who underwent PN or RN. However, many cases in real clinic situation had normal contralateral kidney, therefore renal functional recovery might be mainly due to contralateral kidney enlargement. Actually the increasing rate of kidney volume after RN was significantly larger than PN (9). However there were few studies about renal volumetric correlation analysis according to endophytic degree. In the present study, we investigated renal functional outcomes after PN or RN according to endophytic degree using three dimentional reconstructive kidney volumetrics measured by computed tomography (CT) image, and we intended to provide appropriate tumor size criteria of entire endophytic tumor during PN.

## MATERIALS AND METHODS

### Study population

Our prospectively maintained institutional kidney center database, approved by our Institutional Review Board, was queried to identify all patients from December 2000 to September 2012 undergoing PN or RN with available cross-sectional imaging by CT for assessment. Among them, the patients who had solitary kidney, chronic kidney insufficiency (Modification of Diet in Renal Disease (MDRD) glomerular filtration rate (GFR) <60mL/min/1.73m^2^) and experience of previous kidney surgery were excluded. Patients who had positive surgical margin after PN were also excluded to reduce bias of tumor effect. And we only enrolled the patients with clear cell type RCC after nephrectomy. Accordingly, 214 patients who underwent PN and 220 patients who underwent RN were included. All patients included in this study were from South Korean and resided into South Korea. All surgeries were performed by the single surgeon who were specialist in kidney cancer and had a lot experience with PN or RN over hundreds of cases before these series. All PN were conducted under cold ischemia and open method; all RN were also performed via open methods. Surgical technique for open PN introduced previously was applied in all patients (10).

### Evaluation and kidney volume measurement

Preoperative CT imaging was reviewed in the axial and coronal planes, and a RENAL nephrometry score (NS) was assigned to all identified lesions, as described by Kutikov and Uzzo (11). The NS was categorized as low (4-6 points), moderate (7-9 points) or high (10-12 points) complexity. Tumor endophytic degree was defined along with E score (1, 2, and 3) of NS system. The E score of NS system assigned a point from 1 to 3 according to endophytic nature of the tumor (≥50% exophytic, <50% exophytic or endophytic, respectively). Kidney volume analysis was performed via previous reported methods (9). The kidney volume was measured before and after surgery at 6 months using CT (Somatom Plus 4;Siemens Medical Systems, Forchheim, Germany) with the standard clinical abdominopelvic imaging protocol. Venous scans of entire abdomens were performed with a 60-s delay after starting the 2mL/kg i.v. injection of iodinated contrast agent through an antecubital vein. All axial images were transferred to a workstation running personal computer based software (Rapidia; Infi nitt Co. Ltd, Seoul, Korea), which has been used in previous studies (9, 12). The kidney volume was calculated by summing all the volumes within the normally functioning tissue, excluding tumor tissue or non-enhanced areas in a delayed CT image with a slice thickness of 5mm. Renal volumes were independently measured by three urologists who were blinded to patient characteristics, and final volumes were calculated by averaging the three volumes. The GFR was measured by MDRD equation (13).

### Statistical analysis

Demographic and clinical characteristics were compared between PN and RN. Continuous variables were analyzed by Wilcoxon tests, and categorical variables were examined by chi-square analyses. The renal functional outcomes before and after each nephrectomy according to tumoral endophytic degree were also compared and the ipsilateral and contralateral kidney volume after PN and RN were measured , compared and stratified by degree of endophytic nature. In a sub-analysis, in case of tumor size above 4cm (not clinical T1a), renal functional outcomes and kidney volumetrics were investigated. Intra- and postoperative complications were stratified using the Clavien–Dindo classification system and compared according to surgical methods (14). The prolonged bleeding and hematuria were defined as persistent symptoms 2 weeks after surgery. Statistical analyses were carried out using SPSS version 15.0 software (Statistical Package for Social Sciences™, Chicago, IL, USA). Two-tailed null hypotheses of no difference were rejected if p-values were less than 0.05.

## RESULTS

Demographic and tumor characteristics are summarized in Table-1. The mean age of PN subjects was 53.5 years and of RN subjects was 56.1 years. The mean tumor size was larger in RN group than PN group (7.45 versus 4.04cm, p<0.001); there were more high complexity tumor in RN than PN according to RENAL nephrometry system (61.8% versus 11.3%, p<0.001).

**Table 1:** Descriptive characteristics according to surgical methods.

Variables	Partial nephrectomy	Radical nephrectomy	p-value
N	214	220	
Age (years)±SD	53.52±13.09	56.06±13.83	0.166
**Gender (%)**			0.893
Male	130 (60.7)	158 (71.8)	
Female	84 (39.3)	62 (28.2)	
Body mass index, kg/m^2^±SD	24.8±5.14	24.2±5.74	0.138
**History of hypertension (%)**			0.287
Yes	70 (32.7)	82 (37.3)	
No	144 (67.3)	138 (62.7)	
**History of diabetes (%)**			0.369
Yes	24 (11.2)	30 (13.6)	
No	190 (88.8)	190 (86.4)	
**History of smoking (%)**			0.530
Yes	37 (17.3)	42 (19.1)	
No	177 (82.7)	178 (80.9)	
**ASA score**			0.280
1-3	209 (97.7)	213 (96.8)	
>3	5 (2.3)	7 (3.2)	
Tumor size (cm)±SD	4.04±3.56	7.45±3.78	<0.001
**R.E.N.A.L. nephrometry score, n (%)**			<0.001
Low (4-6)	40 (18.7)	8 (3.6)	
Intermediate (7-9)	150 (70.0)	76 (34.5)	
High (10-12)	24 (11.3)	136 (61.8)	

**SD=** standard deviation; **ASA=** American Society of Anesthesiologists

Mean cold ischemic time was 44.5 min under cold ischemia (Table-2). There were no significant differences with respect to age, gender, body mass index and medical history. Mean preoperative renal function was higher in PN group than RN group (GFR 80.56 versus 70.57mL/min/1.73m^2^, p<0.001). Postoperative complications occurred in 20 patients of PN group and in 12 of RN group; severe complication which needed intervention (Clavien III and IV) was registered in 2 cases in PN group and 1 in RN group. There was no postoperative mortality in both groups.

**Table 2:** Perioperative outcomes characteristics according to surgical methods.

Variables	Partial nephrectomy	Radical nephrectomy	p-value
N	214	220	
Mean cold ischemic time (min)±SD	44.52±16.70	0	-
Mean preoperative serum creatinine (mg/dL)±SD	1.04±0.36	1.28±2.44	0.008
Mean preoperative MDRD GFR (mL/min/1.73m^2^)±SD	80.56±17.27	70.57±39.48	<0.001
Mean operation time (min)±SD	183.4	175.6	0.881
Mean estimated blood loss (cc)±SD	263.7±144.9	310.3±92.7	0.731
Postoperative complications (%)	20 (9.3)	12 (5.5)	0.984
Clavien classification 1-2	16 (7.5)	10 (4.5)	0.176
Clavien classification 3-4	4 (1.9)	2 (0.9)	0.112
Prolonged ileus	4 (1.9)	7 (3.2)	0.217
Wound problem	5 (2.3)	4 (1.8)	0.495
Urine leakage necessary stent insertion	3 (1.4)	0 (0.0)	-
Prolonged bleeding	5 (2.3)	1 (0.5)	0.083
Prolonged hematuria	3 (1.4)	0 (0.0)	-

**MDRD=** modification of diet in renal disease; **GFR=** glomerular filtration rate

**Figure 1:**
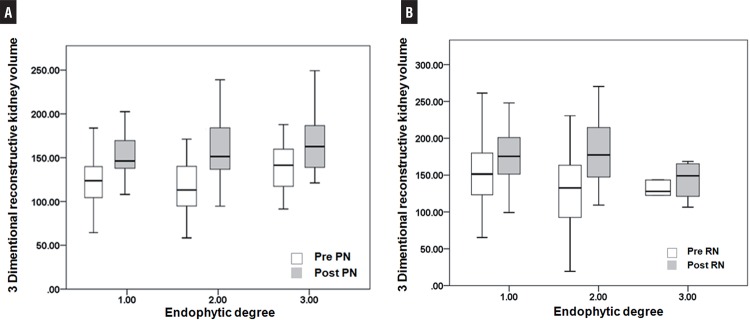
Preoperative and postoperative contralateral kidney volume (non-operated kidney) measured by three dimentional reconstructive method according to endophytic degree after PN (A) and after RN (B).

As shown in Table-3, endophytic degree 1 was noted in 104 patients in PN and 70 in RN groups. Among subgroup endophytic degree 1, preoperative renal function was similar between both groups, however postoperative renal function was better preserved in PN group (GFR PN:76.42 versus RN:55.53mL/min/1.73m^2^, p<0.001). Contralateral kidney (non-surgery kidney) volume which was measured 6 months after nephrectomy was significantly enlarged in RN group than in PN group (RN:169.64 versus PN:149.98mL, p=0.006). Mean volume increase rate in contralateral kidney was also higher in RN group than in PN group (17.9% versus 3.0%). Among endophytic degree 2 groups, postoperative renal function also better preserved in PN group than RN group (GFR PN:79.68 versus RN:51.99mL/min/1.73m^2^, p<0.001) and contralateral kidney volume was larger after nephrectomy in RN group (RN:173.92 versus PN:160.18, p=0.038). Mean volume increase rate in contralateral kidney among endophytic degree 2 group was also higher in RN group than in PN group (24.2% versus 11.8%). However, among endophytic degree 3 group, renal function decreased in both groups and contralateral kidney volume was also similar between both groups. Postoperative renal function (GFR PN: 60.92 versus RN:57.02mL/min/1.73m^2^, p=0.124) and contralateral kidney volume (PN: 138.16 to 159.64 versus RN: 138.65 to 168.04mL, p=0.416) were similar in patients with fully endophytic tumors after PN and RN. Figure-1 shows preoperative and postoperative contralateral kidney volume according to endophytic degree. There were significant increase disparity between PN and RN in cases of endophytic degrees 1 and 2, however similar increase was observed in cases of endophytic degree 3 renal tumor.

**Table 3:** Renal functional outcomes and kidney volumetric results according to endophytic degree among patients who underwent nephrectomy.

Variables	Partial nephrectomy	Radical nephrectomy	p-value
Endophytic degree 1, n (%)	104 (59.8)	70 (40.2)	
Preoperative parameters			
Preoperative serum creatinine (mg/dL)±SD	1.10±0.45	1.15±2.29	0.415
Preoperative MDRD-GFR (mL/min/1.73m^2^)±SD	73.29±19.45	68.53±40.22	0.303
Contralateral kidney volume (mL)±SD	145.55±33.56	143.93±46.93	0.677
Ipsilateral kidney volume (mL)±SD	154.73±35.58	145.07±70.02	0.458
Posteropative parameters			
Postperative serum creatinine (mg/dL)±SD	1.07±0.41	1.49±1.76	0.009
Postperative MDRD-GFR (mL/min/1.73m^2^)±SD	76.42±24.20	55.53±24.11	<0.001
Contralateral kidney volume (mL)±SD	149.98±34.14	169.64±51.01	0.006
Increasing rate of contralateral kidney	3.0%	17.9%	<0.001
Ipsilateral kidney volume (mL)±SD	122.24±33.04	0	
Endophytic degree 2, n (%)	86 (43.9)	110 (56.1)	
Preoperative parameters			
Preoperative serum creatinine (mg/dL)±SD	0.98±0.25	1.22±2.30	0.135
Preoperative MDRD-GFR (mL/min/1.73m^2^)±SD	78.70±15.04	68.03±39.64	<0.001
Contralateral kidney volume (mL)±SD	143.18±38.30	140.03±58.86	0.782
Ipsilateral kidney volume (mL)±SD	147.84±35.54	133.33±108.30	0.425
Posteropative parameters			
Postperative serum creatinine (mg/dL)±SD	0.99±0.25	1.79±1.85	0.006
Postperative MDRD-GFR (mL/min/1.73m^2^)±SD	79.68±16.91	51.99±17.13	<0.001
Contralateral kidney volume (mL)±SD	160.18±32.84	173.92±49.05	0.038
Increasing rate of contralateral kidney	11.8%	24.2%	0.021
Ipsilateral kidney volume (mL)±SD	125.54±30.86	0	
Endophytic degree 3, n (%)	24 (37.5)	40 (62.5)	
Preoperative parameters			
Preoperative serum creatinine (mg/dL)±SD	1.27±0.27	1.48±2.25	0.250
Preoperative MDRD-GFR (mL/min/1.73m^2^)±SD	68.37±14.35	61.91±34.14	<0.001
Contralateral kidney volume (mL)±SD	138.16±37.01	138.65±36.34	0.370
Ipsilateral kidney volume (mL)±SD	115.60±58.36	116.56±44.53	0.854
Posteropative parameters			
Postperative serum creatinine (mg/dL)±SD	1.69±0.25	1.75±3.87	0.206
Postperative MDRD-GFR (mL/min/1.73m^2^)±SD	60.92±14.35	57.02±19.36	0.124
Contralateral kidney volume (mL)±SD	159.64±43.55	168.04±69.38	0.416
Increasing rate of contralateral kidney	15.5%	21.2%	0.184
Ipsilateral kidney volume (mL)±SD	113.50±43.41	0	

**MDRD=** modification of diet in renal disease; **GFR=** glomerular filtration rate

Subgroup analysis of subjects with tumor size above 4cm is shown in Table-4. Among endophytic degree 1 and 2 groups, there was significant better renal functional preservation in PN group than RN group along with significant contralateral kidney enlargement in RN group than PN group. However among endophytic degree 3 group, there was similar postoperative renal function and similar enlargement of contralateral kidney between both groups.

**Table 4:** Renal functional outcomes and kidney volumetrics results after partial or radical nephrectomy in renal tumor above 4cm.

Endophytic degree	1			2			3		
Surgical methods	PN	RN	P-value	PN	RN	p-value	PN	RN	p-value
Pre-operative parameters									
Serum creatinine (mg/dL)±SD	1.18±0.63	1.09±0.32	0.437	0.98±0.11	1.16±2.31	0.322	1.20±0.01	1.33±0.25	0.528
MDRD-GFR (mL/min/1.73m2)±SD	70.48±18.19	75.27±39.67	0.120	78.75±8.84	66.84±40.37	0.641	66.02±12.41	61.86±46.64	0.405
Contralateral kidney volume (mL)±SD	136.25±36.43	154.81±47.32	0.183	155.97±18.02	147.48±52.41	0.784	168.41±22.40	172.20±27.40	0.617
Ipsilateral kidney volume (mL)±SD	151.13±34.77	144.97±71.07	0.675	163.96±23.34	139.46±109.94	0.626	113.25±14.21	118.73±32.93	0.825
Post-operative parameters									
Serum creatinine (mg/dL)±SD	1.22±0.58	1.80±1.78	0.146	1.06±0.17	1.42±0.47	0.095	1.10±0.17	1.86±0.90	0.339
MDRD-GFR (mL/min/1.73m2)±SD	65.48±24.06	55.47±24.38	**0.027**	74.19±7.57	53.98±14.68	**0.004**	59.08±14.83	41.44±15.69	0.228
Contralateral kidney volume (mL)±SD	135.42±32.69	170.53±51.44	**0.039**	162.70±32.40	178.76±41.96	**0.019**	176.14±52.14	185.90±31.41	0.191
Ipsilateral kidney volume (mL)±SD	107.32±34.98	0		125.88±46.59			88.30±13.43		

**MDRD=** modification of diet in renal disease; **GFR=** glomerular filtration rate; **PN=** partial nephrectomy; **RN=** radical nephrectomy

**Bold face=** significant association p<0.05

## DISCUSSION

In the current study, we observed that PN better preserved renal function than RN in cases of mild and moderate exophytic renal masses despite of significant increase of contralateral kidney enlargement of RN than PN. However in cases of entire endophytic tumor, especially above 4cm, renal functional outcomes were similar after PN or RN; contralateral kidney enlargement was also similar between both surgical methods.

Rate of PN continues to increase worldwide based on the growing literature supporting its renal function benefits relative to RN. According to 2006 SEER cancer registry, 45% of patients with small renal mass underwent PN, however recent contemporary reports from single major referral centers describe a PN rate of up to 89% for tumors 4cm or less (15, 16). And currently, greater understanding of the biological heterogeneity of small renal masses and increased awareness of the risks of chronic kidney disease have led to greater use of PN for larger and more complex tumors (15). Lane et al. (15) showed 54% of clinical T1b tumors were treated with PN between 2004 and 2012 in a multicenter study. These results might have been originated from renal function preservation concerns: PN could preserve renal parenchymal tissue in some amount. Actually RN had previously been found to be associated with a greater risk of de novo chronic renal failure than PN (17, 18). However in cases with large tumor size and high tumor endophytic degree, ischemic time and perioperative complications should be increased, therefore NS was introduced (11). Many studies confirmed its usefulness for predicting surgery type and renal functional outcome (19, 20).

Another predictor of renal function after nephrectomy was kidney volume. Kidney volume is an important parameter of renal function in the evaluation and follow-up of patients with end stage renal disease, polycystic kidney and transplanted kidney (21, 22). In these diseases, change of kidney volume became a reliable parameter of disease progression and renal function. By measuring kidney volume with traditional ultrasound, using the dimensions of the 3 orthogonal axes to the ellipsoid formula, there is some error and poor reproducibility (23). However, along with recent improvement of imaging technique, relative accurate kidney volume measurement by CT or magnetic resonance imaging was introduced and these methods can be applied after PN. Previous study to investigate kidney volume in 133 patients showed preoperative kidney volume was independent predictor of postoperative GFR in PN or RN patients (9). Simmons et al. (24) also reported that kidney volume measured by cylindrical volume ratio method was well preserved along with NS and indicator of renal function. Gong et al. (25) also showed that kidney volume correlated well with renal function in 539 normal patients.

However, in real clinical situation of PN, we should consider contralateral kidney change. Traditionally RN was thought to be an acceptable surgery due to compensatory recovery of renal function by contralateral kidney. Kidney transplantation could be also acceptable by this reason. Anderson et al. (26) examined renal function after donor nephrectomy and noted that compensatory hypertrophy was completed 1 week after surgery and that the effective renal plasma flow had increased by 32.5% in the residual kidney. They reported that the effective renal plasma flow recorded 10 years after surgery was still greater than the preoperative level and that the percentage of decrease in the effective renal plasma flow during the 10-year postoperative follow-up did not differ from that of normal controls. Contralateral kidney volume and function after PN were significant higher than after RN. Jeon et al. (9) showed the volume of normal side kidney increased 127.2 to 138.8mL after PN, however normal side kidney after RN increased 142.4 to 166.0mL. One study about effective renal plasma flow using dynamic scintigraphy showed that renal flow increased 3.8% after RN and 0.1% after PN (25). In our study, contralateral kidney volume which was measured 6 months after nephrectomy was significantly more enlarged in RN than PN group in exophytic degrees 1 and 2. Among endophytic degree 3 tumor, which was entirely endophytic mass, similar kidney volume increase after surgery between PN and RN was observed due to poor functional preservation of operative kidney. It might be considered as evidence that remnant operated kidney after PN in entirely endophytic tumor had not good functional contribution to the total kidney function. Especially large masses above 4cm with entirely endophytic feature should consider RN as the method of choice for nephrectomy due to not only less benefit of remnant kidney function but also harmfulness of high complication.

Generally authors also agree with the concept that PN could preserve renal function well, however in cases of entire endophytic tumor we reconsider which choice of surgical methods will be better. Central tumor site is associated with increased complication rates, collecting system entry and ischemia time (27). Tumor endophytic percent is associated with an increased complication rate (28). Another reason is that kidney volume before nephrectomy in entirely endophytic mass was already increased, meaning that unilateral renal function was already decreased in mass containing kidney. After extraction of central tumor, remnant kidney might not have significant function.

Our study has several limitations. There was sample number disparity according to endophytic degree and surgical methods, despite the consecutive nature of data from our kidney center database. Current many retrospective studies had selectional bias that more complex and large tumors are usually extracted by RN. Also, a small numbers of patients were enrolled in endophytic degree 3 group and these patients had relative low renal function at baseline. Second, preoperative renal scintigraphy was not obtained in the present study and we could not calculate a single GFR for the normal side kidney. Previous study had reported a strong correlation between kidney volume and renal scintigraphy (29). And we could not adjust cold ischemic time in PN group due to its retrospective nature. Furthermore, another study had provided evidence indicating that split renal function could be calculated by measuring the kidney volume (30). Unfortunately, we could not access this method due to its retrospective nature.

## CONCLUSIONS

PN preserved renal function better than RN in relative exophytic masses, however in cases of entirely endophytic mass, renal functional outcome after PN was similar to RN especially in large size tumors. Contralateral kidney volume enlargement was significantly increased after RN than PN, except in entirely endophytic masses. Therefore we should consider RN as the preferential surgical option in entirely endophytic masses with large size to reduce PN related complications.
